# Polyunsaturated fatty acids, *APOE* genotypes, and dementia incidence and mortality among hypertensive adults

**DOI:** 10.1016/j.tjpad.2025.100297

**Published:** 2025-07-22

**Authors:** Yubo Zhang, Jindi Li, Shaohui Liu, Quanhong Chen, Xuexiu Wang, Sisi He, Yadong Wei, Yunfeng Zou, Yunan Xu, Lijun Wang, Hao Chen

**Affiliations:** a Department of Occupational and Environmental Health, School of Public Health, Guangxi Medical University, Nanning 530021, China; b Department of Toxicology, School of Public Health, Guangxi Medical University, Nanning 530021, China; c Guangxi Key Laboratory of Environment and Health Research, Guangxi Medical University, Nanning, Guangxi 530021, China; d Guangxi Colleges and Universities Key Laboratory of Prevention and Control of Highly Prevalent Diseases, Guangxi Medical University, Nanning 530021, China; e Department of Medical Research, The First Affiliated Hospital of Guangxi Medical University, Nanning 530021, China; f Department of Epidemiology and Biostatistics, School of Public Health, Guangxi Medical University, Nanning, Guangxi 530021, China

**Keywords:** UK biobank, Hypertension, Polyunsaturated fatty acids, Dementia, Polygenic risk scores, *APOE* genotypes

## Abstract

**Background:**

Individuals with hypertension have an elevated risk of dementia. The potential protective effects of dietary polyunsaturated fatty acids (PUFAs) against dementia remain unclear. In this study, we investigate associations between blood PUFA levels and dementia outcomes, while considering the genetic predisposition among hypertensive adults.

**Methods:**

We employed data from UK Biobank and a prospective cohort of 123,235 hypertensive participants aged 40–69 years were included for the analysis (2006–2022). Cox proportional hazards models adjusting for covariates were applied to assess the associations of blood levels of docosahexaenoic acid (DHA), N3FA, N6FA, linoleic acid (LA), total PUFA, and the N6FA/N3FA ratio with incident dementia, dementia mortality, and all-cause mortality. The analyses were also stratified by polygenic risk scores (PRS) or *APOE* genotypes.

**Results:**

Higher levels of DHA (HR 0.41, 95 % CI 0.27–0.62), N3FA, LA, N6FA, and total PUFA were associated with significantly reduced dementia incidence (*P* < 0.001). In contrast, a higher N6FA/N3FA ratio was linked to increased dementia risk. Similar trends were observed for mortality. *APOE* genotypes, rather than PRS, modified PUFA–dementia associations: individuals with low-to-moderate *APOE* risk showed greater protective effects of high PUFA levels compared to those with high-risk genotypes.

**Conclusions:**

Among hypertensive adults, higher PUFA levels are associated with reduced risks of dementia and mortality. An imbalanced N6FA/N3FA ratio increases risk, while *APOE* genotypes significantly modify PUFA-related dementia outcomes.

## 1 Introduction

Hypertension is a highly prevalent chronic condition globally, affecting over 1.13 billion adults worldwide [1]. Despite advancements in methodologies associated with hypertension detection and management, inadequate blood pressure control remains a significant clinical and public health challenge, leading to increased incidence, mortality, and healthcare burden associated with cardiovascular, nephrological, and neurological complications [2,3].

Dementia is a condition lacking effective treatment and represents a severe and escalating public health crisis. Approximately, 7 million individuals aged 65 and older in the United States were diagnosed with Alzheimer’s disease (AD), the most common form of dementia, and the fifth leading cause of death in the country [[4], [5], [6]]. Growing epidemiological, clinical, and experimental evidence suggested an association between hypertensive status and dementia incidence [7]. For instance, a meta-analysis indicated that hypertensive individuals had 1.25 times higher risk of developing dementia compared to normotensive individuals [8]. Possible mechanisms underlying elevated dementia risk caused by hypertension include cerebrovascular damage, amyloid deposition, oxidative stress and chronic inflammation affecting neurons and blood vessels [9]. As a major late-life dementia risk factor, it's crucial to find interventional strategies to prevent dementia in those with high blood pressure.

Previous studies suggested that polyunsaturated fatty acids (PUFAs), a type of dietary fat with multiple carbon-carbon double bonds, may lower dementia risk. Although several epidemiological studies have reported an association between consumption of omega-3 polyunsaturated fatty acids (N3FA) and a reduced risk of cognitive decline and dementia, a few indicated null associations [10,11]. Similarly, increased dietary intake of omega-6 polyunsaturated fatty acids (N6FA), such as γ-Linolenic Acid (GLA), has shown protective effects against neurological disorders [12]. While both N6FA and N3FA play crucial roles in maintaining cell membrane integrity, regulating inflammation, and supporting neurological health [13]. The N6FA/N3FA ratio may dictate the modulation of dementia risk more than the absolute levels of either fatty acid. This possibility stems from the duality of N6FA metabolism, while certain N6FA derivatives, such as prostaglandin E₂ and leukotriene B₄ from arachidonic acid (AA, N-6), can exert pro-inflammatory effects that potentially increase dementia risk [14,15], other N6FA metabolites, such as docosapentaenoic acid (DPA N-6) from linoleic acid, demonstrate neuroprotective properties [16,17]. These findings present seemingly contradictory observations regarding PUFA's protective effects against dementia, warranting further investigations that account for the complex metabolic interplay.

Genetic susceptibility may significantly interact with PUFAs on dementia risk. There are two aspects of genetic predisposition related to either PUFA utilization or dementia onset [18,19]. Genetic factors associated with genetic polymorphisms in *FADS1/FADS2* (fatty acid desaturase 1 and 2) affect the activity of the Δ5 and Δ6 desaturase enzymes [20], thereby influencing absorption and utilization of PUFAs. Additionally, the ε4 allele of the *APOE* (apolipoprotein E) gene is the most well-established genetic risk factor for late-onset AD [21]. Beyond *APOE*, genome-wide association studies (GWASs) show that cognitively normal individuals had four times more protective gene enrichment than AD patients [22]. Polygenic risk scores (PRS) aggregate these variants, providing a comprehensive measure of genetic predisposition [[23], [24], [25]]. Therefore, understanding the interplay between genetic predisposition associated with PUFA utilization and dementia onset may reveal optimal strategies for reducing dementia risk.

In this study, we employed data from the UK Biobank and aim to investigate the associations of blood levels of PUFAs with dementia incidence and mortality among hypertensive participants, while considering genetic susceptibility associated PUFAs utilization and dementia onset.

## 2 Materials and methods

### 2.1 Study population

The UK Biobank is a prospective, population-based cohort that recruited approximately 500,000 individuals aged 40 to 69 years between 2006 and 2010 across 22 centers in the UK [26]. Baseline data included questionnaires on socio-demographic characteristics, diet, and lifestyle factors, as well as biological samples, and physical measurements. Longitudinal monitoring was realized through in-person assessments and electronic medical records. The UK Biobank obtained ethical approval (Ref. 11/NW/0382) and all participants provided electronic informed consent. This research was conducted using data from the UK Biobank Resource under application number [Project ID: 97,753].

As shown in Fig. S1, 269,462 hypertensive patients were identified by meeting at least one of the following criteria: systolic blood pressure (SBP) ≥ 140 mmHg and/or diastolic blood pressure (DBP) ≥ 90 mmHg at baseline; use of antihypertensive medications; self-reported hypertension; or a formal diagnosis from a healthcare professional. Exclusions were made for those lacking fatty acid measurements (*n* = 145,791), pregnant individuals (*n* = 337), and those with baseline dementia (*n* = 89), resulting in a final sample of 123,235 hypertensive patients. A subgroup analysis was conducted on 25,902 participants with PRS data associated with PUFA or dementia onset to examine their interactions.

### 2.2 PUFA assessment

Blood samples were collected during the initial (2006–2010) and first follow-up (2012–2013) phases. A total of 251 metabolic biomarkers—including lipoproteins, fatty acids, the composition of fatty acids, and various low molecular weight metabolites—were measured among approximately 280,000 participants using a nuclear magnetic resonance (NMR) analysis platform (Nightingale Health Ltd) [27]. Our analysis included plasma concentrations of N3FA, N6FA, N6FA/N3FA ratio, docosahexaenoic acid (DHA) and linoleic acid (LA). Total PUFA were calculated by summing N3FA and N6FA as a proxy as well as the N6FA/N3FA ratio [28]. The UK Biobank database did not provide data on eicosapentaenoic acid (EPA).

### 2.3 Ascertainment of health outcomes

Incident cases of dementia were ascertained through linkage with hospital inpatient records (Hospital Episode Statistics for England, Morbidity Records for Scotland, and the Patient Episode Database for Wales) and death registration data from the National Health Service (NHS) records. Each case was defined according to the International Statistical Classification of Diseases and Related Health Problems, 10th Revision (ICD-10). The primary outcomes of interest in this study were:

(i) **Dementia events**: These included multi-infarct dementia, vascular dementia, unspecified dementia, Alzheimer’s disease (AD) with early onset, AD with late onset, and unspecified AD. The relevant ICD-10 codes for these conditions were F01.1, F01.9, F03, G30.0, G30.1, and G30.9 [27,29].
(ii) **All-cause and AD-related mortality**: Data for all-cause and dementia-specific deaths were collected. Participants who were alive at the end of follow-up were censored on December 19, 2022. Follow-up time was calculated in person-years from the date of enrollment until the occurrence of either an AD diagnosis or death, whichever came first. The ICD-10 codes for AD-related deaths were G30.0, G30.1, and G30.9.

### 2.4 Assessment of genetic susceptibility

The UKB included PRS data for 53 diseases and quantitative traits, generated using a Bayesian approach applied to meta-analyzed (when possible, ancestry specific) summary statistics data obtained entirely from external GWAS [30]. Since no PRS scores for dementia were available, we used PRS for AD as a proxy of dementia onset. Additionally, we obtained PRS for PUFA, N3FA, N6FA and DHA. The PRS were normalized to a mean of 0 and a standard deviation (SD) of 1. Genetic principal components (PCs) were derived from genotyping data [23,31]. *APOE* status was determined by rs429358 and rs7412, with ε4/ε4 or ε3/ε4 indicating high dementia risk, ε2/ε3 or ε2/ε2 indicating low risk, and ε3/ε3 or ε2/ε4 representing moderate risk [32,33]. Due to the low number of deaths in the low-risk population, participants were dichotomized into low-to-moderate risk (*n* = 90,879) and high risk (*n* = 32,356) groups based on *APOE* phenotypes.

### 2.5 Covariates

The potential confounding variables included sociodemographic factors (age, sex, ethnicity, and education), lifestyle habits (smoking status, drinking status), body mass index (BMI), and medications (antihypertensive drug use, lipid lowering medication, and aspirin use). Education was categorized as high school or below or above high school. International Physical Activity Questionnaire was employed to assess the metabolic equivalent of task (MET), and physical activity level was assessed by MET hours per week [34]. Cardiovascular diseases (CVD) and diabetes were diagnosed by health professionals (yes or no). *APOE* genotype status was also included as a covariate when evaluating the associations between PUFA and dementia risk.

### 2.6 Statistical analysis

Continuous variables are presented as mean ± standard deviation (SD), and categorical variables as count (percentage). Cox proportional hazards regression models were employed to assess the relationship of blood PUFA concentrations (as both continuous and categorical variables) with dementia incidence and mortality risk, adjusting for age, sex, race, smoking and alcohol consumption status, educational attainment, household income, Townsend Deprivation Index, physical activity, body mass index (BMI), antihypertensive medication use, serum cholesterol levels, chronic kidney disease, diabetes, and *APOE* genotypes. Adjusted hazard ratios (aHRs) with their 95 % confidence intervals (CIs) were reported.

To evaluate possible modification of genetic susceptibility on the correlations between PUFAs and dementia incidence and mortality risk, we included interaction terms between PUFA parameters and respective PRS for PUFA (PUFA-PRS) or AD (AD-PRS). Because *APOE* genotypes is a key genetic contributor of dementia occurrence, we examined the modification of *APOE* status on the associations between PUFA and dementia risk. Covariate variables mentioned above were also adjusted in the model.

We further conducted several sensitivity analyses. Firstly, a subgroup analysis was conducted to investigate sex-specific associations between PUFAs and dementia incidence and mortality. Secondly, to account for time-varying effects of PUFA measurements during the follow-up, we employed a time-dependent cox regression model to assess the associations between two PUFA measurements and dementia risk (the number of participants with second follow-up PUFA data were 7960). To mitigate reverse causality, individuals with secondary follow-up data who experienced a dementia event prior to the second PUFAs measurement (conducted in 2012–2013) were excluded from the analysis.

All statistical analyses were performed using R Studio version 4.4.1, with statistical significance set at two-sided *P* values <0.05.

## 3 Results

### 3.1 Participant characteristics

As shown in Table 1, among the 123,235 participants, more than half of the participants were over 60 years old (55.2 %) and had completed high school education or below (90.2 %). Lifestyle factors showed only 8.9 % participants were current smokers, 91.9 % consumed alcohol regularly, 80.6 % engaged in moderate to high levels of physical activity, and 61.9 % reported sleeping less than eight hours per night. All participants were hypertensive, but a significant majority (82.4 %) were not taking antihypertensive medication. Diabetes were reported among 8.0 % participants while 52.3 % had cardiovascular diseases during the follow-up. Blood levels of DHA, N3FA, N6FA, N6FA/N3FA, LA, and total PUFA were 0.23 mmol/L, 0.51 mmol/L, 4.48 mmol/L, 8.72, 3.40 mmol/L, and 5.02 mmol/L, respectively (Table 2).

**Table 1:** Participant characteristics.

Variables	Total (*n* = 123,235)
Age, years, n ( %)	
<60	55,255 (44.8)
≥60	67,980 (55.2)
Sex, n ( %)	
Female	59,022 (47.9)
Male	64,213 (52.1)
Race/ethnicity, n ( %)	
White	116,021 (94.1)
Other races	7214 (5.9)
Education level, n ( %)	
High school or below	111,099 (90.2)
Above high school	12,136 (9.8)
Annual household income, n ( %)	
<£31,000	64,222 (52.1)
≥£31,000	59,013 (47.9)
Smoking status, n ( %)	
Never	64,852 (53.6)
Previous	47,391 (38.5)
Current	10,992 (8.9)
Alcohol use in the past year, n ( %)	
Never	5647 (4.6)
Previous	4392 (3.5)
Current	113,196 (91.9)
Physical activity level, n ( %)	
Low	23,907 (19.4)
Moderate	49,386 (40.1)
High	49,942 (40.5)
BMI, kg/m², n ( %)	
<18.5	323 (0.3)
18.5–24.9	28,228 (22.8)
25–29.9	55,037 (44.7)
≥30	39,647 (32.2)
Diabetes, n ( %)	
Yes	9821 (8.0)
No	113,414 (92.0)
Taking antihypertensive medication, n ( %)	
Yes	21,643 (17.6)
No	101,592 (82.4)
Sleep duration, n ( %)	
<8	76,516 (61.9)
≥8	46,719 (38.1)
Cardiovascular disease, n ( %)	
Yes	64,496 (52.3)
No	58,739 (47.7)
Townsend deprivation index, Median (IQR)	−2.23 (−3.68,0.39)
*APOE* genotypes, n ( %)	
Low to moderate	90,879 (73.7)
High	32,356 (26.3)

Note: Categorical variables were expressed as number (n) and weighted percent ( %) of the participants. Continuous variables were expressed as weighted median and interquartile range (IQR). All statistics were adjusted with survey effects. *APOE*: apolipoprotein E, BMI: body mass index.

**Table 2:** Blood levels of polyunsaturated fatty acids (PUFAs) among hypertensive participants (*n* = 123,235) in the UK Biobank.

Fatty acids	Median (IQR)
PUFA, mmol	5.02 (4.49,5.58)
N3FA, mmol	0.51 (0.39,0.67)
N6FA, mmol	4.48 (4.03,4.96)
N6FA/N3FA ratio	8.72 (6.95,11.04)
DHA, mmol	0.23 (0.18,0.28)
LA, mmol	3.40 (2.96,3.88)

Note: Blood levels of polyunsaturated fatty acids were assessed by a nuclear magnetic resonance methodology and data were expressed as median with interquartile range (IQR). DHA: docosahexaenoic acid, LA: linoleic acid, N3FA: omega-3 polyunsaturated fatty acids, N6FA: omega-6 polyunsaturated fatty acids, PUFA: polyunsaturated fatty acids.

### 3.2 Associations of blood level of PUFAs with dementia and mortality risk

**Fig. 1:**
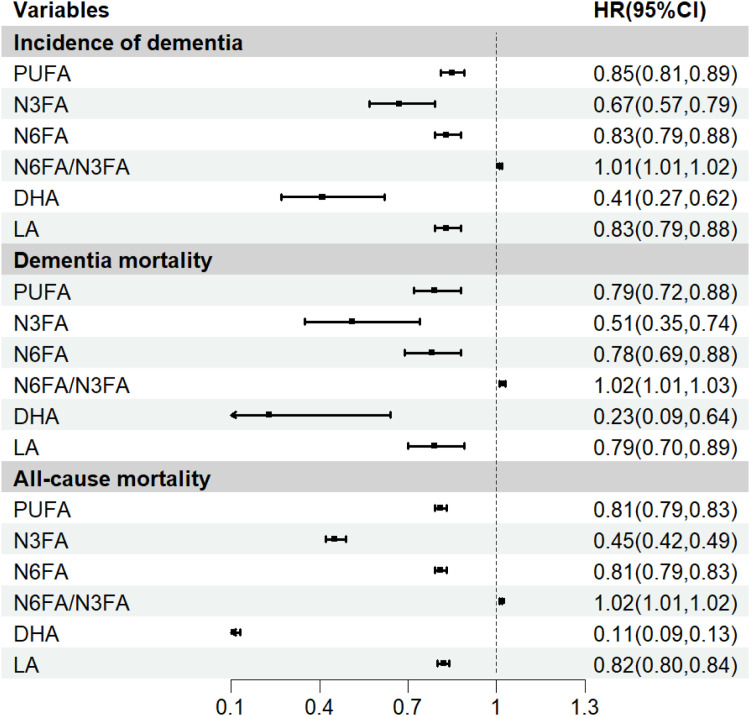
The associations of PUFAs with dementia and mortality risk among hypertensive participants in the UK Biobank. Note: This model utilized a cox proportional hazards model. The model was adjusted for covariates including age, sex/gender, race/ethnicity, educational level, annual household income, body mass index, smoking status, alcohol use in the past year, physical activity level, diabetes, cardiovascular disease, sleep duration, Townsend deprivation index, taking antihypertensive medicine, and *APOE* genotypes. *APOE*: apolipoprotein E, DHA: docosahexaenoic acid, LA: linoleic acid, N3FA: omega-3 polyunsaturated fatty acids, N6FA: omega-6 polyunsaturated fatty acids, PUFA: polyunsaturated fatty acids.

Participants with elevated blood concentrations of DHA [aHR, 0.41; 95 % CI, (0.27,0.62)], N3FA [0.67, (0.57,0.79)], LA [0.83, (0.79,0.88)], N6FA [0.83, (0.79,0.88)], and total PUFA [0.85, (0.81,0.89)] (*P* < 0.001) were associated lower risk of having dementia. Conversely, individuals with an elevated N6FA/N3FA ratio demonstrated increased risk [1.01, (1.01,1.02)] (Fig. 1). Similarly, hypertensive individuals with higher circulating levels of DHA, N3FA, LA, N6FA and total PUFA showed lower risk of dementia-specific and all-cause mortality (*P* < 0.001). In contrast, elevated N6FA/N3FA ratios were correlated with increased dementia-specific and all-cause mortality (*P* < 0.001) (Fig. 1). When PUFAs were categorized by quartiles, their relationships with dementia incidence, dementia-specific mortality, and all-cause mortality remained consistent with the continuous-variable analysis (Table S1).

### 3.3 Interaction between blood levels of PUFAs and genetic risk on dementia incidence and mortality

Participants were divided into high and low PUFA-PRS groups based on median PRS for DHA, N3FA, N6FA, and PUFA. We observed that blood levels of total PUFA and N6FA were associated with lower risk dementia and mortality risk, regardless of PRS category (Table 3). No significant differences in PUFA-dementia associations were seen between low and high risks groups of PUFA-PRS (Table 3). In low and high AD-PRS groups, the association between N6FA/N3FA ratio and all-cause mortality in the low AD-PRS group was significantly stronger than that in the high AD-PRS group [Low vs. high: 1.02 (1.02, 1.03) vs. 1.01 (1.01, 1.02), *P _interaction_* < 0.05]. However, we did not observe any other significant differences in the association of other PUFA parameters with dementia outcomes between the low and high AD-PRS groups (Table 4).

**Table 3:** Association between the blood levels of PUFA and the incidence and mortality of dementia stratified by genetic risk of PUFAs among hypertensive adults in UK Biobank.

	Hazards ratio [95 %CI]		*P _interaction_*
	Low PUFA-PRS	High PUFA-PRS	
Dementia incidence			
PUFA	0.81(0.70,0.94) **	0.79(0.69,0.90) ***	0.55
N3FA	0.65(0.39,1.10)	0.78(0.48,1.26)	0.70
N6FA	0.74(0.62,0.89) **	0.77(0.66,0.90) ***	0.96
DHA	0.72(0.20,2.51)	0.38(0.10,1.40)	0.53
Dementia mortality			
PUFA	0.79(0.68,0.92) **	0.78(0.68,0.89) ***	0.58
N3FA	0.58(0.34,0.99) *	0.72(0.45,1.18)	0.60
N6FA	0.72(0.60,0.87) ***	0.76(0.65,0.89) ***	0.83
DHA	0.52(0.15,1.87)	0.28(0.07,1.00)	0.48
All-cause mortality			
PUFA	0.81(0.75,0.87) ***	0.88(0.82,0.94) ***	0.40
N3FA	0.50(0.38,0.65) ***	0.53(0.41,0.69) ***	0.72
N6FA	0.80(0.74,0.87) ***	0.90(0.84,0.98) ***	0.11
DHA	0.12(0.06,0.24) ***	0.14(0.07,0.28) ***	0.84

Note: This model utilized the cox proportional hazards model. The model was adjusted for covariates including age, sex/gender, race/ethnicity, educational level, annual household income, body mass index, smoking status, alcohol use in the past year, physical activity level, diabetes, cardiovascular disease, sleep duration, Townsend deprivation index, taking antihypertensive medicine, and *APOE* genotypes. PRS scores for total PUFA, N3FA, N6FA, and DHA were dichotomized based on the median to categorize individuals as having either "low" or "high" genetic risk. *APOE*: apolipoprotein E, DHA: docosahexaenoic acid, LA: linoleic acid, N3FA: omega-3 polyunsaturated fatty acids, N6FA: omega-6 polyunsaturated fatty acids, PUFA: polyunsaturated fatty acids, PRS: polygenic risk scores. * *p* < 0.05; ** *p* < 0.01; *** *p* < 0.001.

**Table 4:** Associations of the blood levels of PUFAs with the incidence and mortality of dementia stratified by genetic risk of dementia or *APOE* status among hypertensive adults.

	Hazards ratio [95 %CI] (*n* = 25,902)		*P _interaction_*	Hazards ratio [95 %CI] (*n* = 123,235)		*P _interaction_*
	Low AD-PRS	High AD-PRS		Low to moderate risk associated *APOE status*	High risk associated *APOE status*	
Dementia incidence						
PUFA	0.78(0.65,0.93) **	0.82(0.73,0.92) ***	0.27	0.74(0.64,0.84) ***	0.90(0.78,1.03)	0.01 **
N3FA	0.66(0.35,1.25)	0.74(0.49,1.11)	0.68	0.61(0.37,0.98) *	0.83(0.51,1.35)	0.19
N6FA	0.74(0.60,0.91) **	0.79(0.69,0.90) ***	0.25	0.69(0.59,0.81) ***	0.88(0.75,1.04)	0.01 **
N6FA/N3FA	1.01(0.98,1.04)	1.01(1.00,1.02)	0.88	1.01(1.00,1.02)	1.01(0.98,1.04)	0.89
DHA	0.39(0.07,2.04)	0.58(0.20,1.65)	0.53	0.25(0.07,0.92) *	0.98(0.29,3.28)	0.04 *
LA	0.75(0.60,0.92) **	0.78(0.68,0.90) ***	0.26	0.68(0.58,0.80) ***	0.89(0.75,1.05)	0.01 **
Dementia mortality						
PUFA	0.76(0.63,0.91) **	0.80(0.71,0.90) ***	0.22	0.57(0.39,0.82) **	0.72(0.51,1.02)	0.26
N3FA	0.62(0.32,1.17)	0.67(0.44,1.02)	0.71	0.14(0.03,0.62) **	0.82(0.27,2.47)	0.05 *
N6FA	0.72(0.59,0.90) **	0.77(0.67,0.90) ***	0.2	0.55(0.36,0.86) **	0.65(0.43,0.98) *	0.46
N6FA/N3FA	1.01(0.99,1.04)	1.01(1.00,1.03) *	0.94	1.03(1.01,1.04) ***	0.96(0.89,1.04)	0.15
DHA	0.30(0.06,0.63)	0.42(0.15,1.23)	0.56	0.01(0.00,0.11) **	1.70(0.13, 22.73)	0.01 **
LA	0.73(0.59,0.90) **	0.77(0.67,0.88) ***	0.21	0.57(0.37,0.89) *	0.66(0.44,0.99) *	0.05
All-cause mortality						
PUFA	0.85(0.80,0.91) ***	0.84(0.79,0.90) ***	0.96	0.83(0.79,0.88) ***	0.89(0.81,0.98) *	0.06
N3FA	0.54(0.42,0.70) ***	0.49(0.38,0.63) ***	0.77	0.50(0.41,0.62) ***	0.55(0.39,0.79) **	0.38
N6FA	0.86(0.79,0.93) ***	0.85(0.78,0.92) ***	0.87	0.83(0.78,0.89) ***	0.91(0.81,1.01)	0.05
N6FA/N3FA	1.02(1.02,1.03) ***	1.01(1.01,1.02) ***	0.01 **	1.01(1.01,1.02) ***	1.03(1.01,1.05) ***	0.14
DHA	0.16(0.08,0.31) ***	0.12(0.06,0.23) ***	0.70	0.12(0.07,0.21) ***	0.17(0.07,0.43) ***	0.32
LA	0.87(0.80,0.94) ***	0.86(0.79,0.93) ***	0.92	0.83(0.78,0.89) ***	0.92(0.83,1.03)	0.03 *

Note: This model utilized the cox proportional hazards model. The model was adjusted for covariates including age, sex/gender, race/ethnicity, educational level, annual household income, body mass index, smoking status, alcohol use in the past year, physical activity level, diabetes, cardiovascular disease, sleep duration, Townsend deprivation index, taking antihypertensive medicine. The interaction term is the multiplication of PUFAs and *APOE* genotypes. The phenotypes of ε4/ε4 or ε3/ε4 represented a high risk of dementia, whereas ε2/ε3 or ε2/ε2 for a low risk, and ε3/ε3 or ε2/ε4 for a normal risk between high and low risk. *APOE*: apolipoprotein E, DHA: docosahexaenoic acid, LA: linoleic acid, N3FA: omega-3 polyunsaturated fatty acids, N6FA: omega-6 polyunsaturated fatty acids, PUFA: polyunsaturated fatty acids, PRS: polygenic risk scores. * *p* < 0.05; ** *p* < 0.01; *** *p* < 0.001.

Individuals carrying low-to-moderate dementia risk associated with *APOE* genotypes demonstrated a significantly lower risk of developing dementia with higher blood levels of PUFA [Low-to-moderate vs. high: 0.74 (0.64,0.84) vs. 0.90 (0.78,1.03)], N6FA [0.69 (0.59,0.81) vs. 0.88 (0.75,1.04)], DHA [0.25 (0.07,0.92) vs. 0.98 (0.29,3.28)], and LA [0.68 (0.58,0.80) vs. 0.89 (0.75,1.05)], compared with those carrying high-risk *APOE* genotypes (*P _interaction_* < 0.05). Furthermore, in the low-to-moderate risk group, the associations of higher blood levels of N3FA [0.14 (0.03, 0.62) vs. 0.82 (0.27, 2.47)] and DHA [0.11 (0.00, 0.11) vs. 1.70 (0.13, 22.73)] with dementia-related mortality were significantly stronger (*P _interaction_* < 0.05) compared to high-risk *APOE* genotypes (Table 4). In addition, the protective associations of LA with all-cause mortality were stronger among the individuals with low-to-moderate-risk *APOE* genotypes than those high-risk *APOE* genotypes [0.83 (0.78, 0.89) vs. 0.92 (0.83, 1.03), *P _interaction_* < 0.05].

### 3.4 Subgroup and sensitivity analysis

Results from the sex-stratified subgroup analyses indicated that the protective associations of PUFA, N3FA, N6FA, DHA, and LA with dementia incidence as well as all-cause mortality were more pronounced in men compared with women (*P* < 0.05) (Table S2). Additionally, a higher N6FA/N3FA ratio was linked to a modestly increased risk of dementia incidence (*P* < 0.05) and all-cause mortality (*P* < 0.001) in men compared to women. To control for potential confounding effects of depression, we included depression status as a covariate in the statistical model and the results remained robust (Fig. S2). To reduce reverse causality, only people who developed dementia or died two years after baseline enrollment were defined as cases, and the results were consistent with the main findings ((Fig. S3). Using time-dependent models, a subset of participants (*n* = 8302) with PUFA measurements at both baseline and the first follow-up were included. The associations between PUFA levels and dementia incidence, dementia-specific mortality, and all-cause mortality remained consistent across time points, confirming the stability of our findings (Table S3).

## 4 Discussion

In this prospective cohort study, we found that blood levels of PUFA were associated with lower risk of dementia incidence, dementia mortality, and all-cause mortality, while higher N6FA/N3FA ratio with elevated risk. Additionally, we observed an interaction between PUFAs and *APOE* genotypes on dementia incidence, dementia mortality, and all-cause mortality. Compare with individuals with high dementia onset risk associated *APOEε4* status, those with low-to-moderate dementia risk due to *APOE* genotype showed a significantly reduced risk of dementia incidence and mortality when they had higher blood levels of PUFAs.

Intake of PUFA, such as DHA, was significantly associated with a lower risk of dementia incidence and mortality. The neuroprotective effects of PUFAs may be mediated through their anti-inflammatory and antioxidant properties. For instance, N3FAs including DHA and EPA may be incorporated into neuronal membrane phospholipids, reducing the release of arachidonic acid and its pro-inflammatory derivatives (e.g., PGE₂) by inhibiting the cyclooxygenase-2 (COX-2) and lipoxygenase (LOX) pathways—mechanisms similar to those of non-steroidal anti-inflammatory drugs (NSAIDs) [35]. DHA also enhances the expression of antioxidant enzymes (e.g., catalase, glutathione peroxidase), mitigates oxidative stress, and its metabolite neuroprotection D1 (NPD1) promotes anti-apoptotic signaling and inhibits caspase activity, thereby reducing neuronal apoptosis [36].

Previous studies have suggested that dietary PUFA may protect against dementia onset and mortality risk [[37], [38], [39], [40]], with majority focusing on individual N3FA or N6FA concentrations, rather than the ratio between the two. Our study showed that a higher N6FA/N3FA ratio exhibited elevated risk with dementia and mortality risk in hypertensive participants, aligning with research linking this ratio to chronic disease development [41]. This effect may stem from the pro-inflammatory effects of N6FA, which can impair cognitive function [[42], [43], [44], [45]] and exacerbate inflammation [46,47]. A balanced N6FA/N3FA ratio, such as a minimum of 8, was correlated with lower cardiovascular disease risk [48]. Thus, monitoring and regulating this ratio could be a key strategy to reduce dementia incidence and mortality.

The bioavailability and utilization of PUFAs can vary among individuals that can limit PUFA ingestion and bioavailability, influenced by genetic factors. For instance, the *Mfsd2a* gene, which encodes a transporter protein for DHA, affects its uptake across the blood-brain barrier. Diminished *Mfsd2a* function impairs DHA uptake and neuroprotection [49]. Additionally, PPARα, a nuclear receptor regulating lipid metabolism and amyloid precursor protein processing, promotes neuronal survival and reduces AD risk [50,51]. Therefore, genetic susceptibility can affect the protective effects of PUFAs on dementia. However, our results showed that PUFA-PRS did not significantly modulate the associations between PUFAs and dementia incidence or mortality, suggesting that health benefits of PUFA are independent of genetic predisposition for their bioavailability and utilization.

We found no significant differences in the associations of DHA, N3FA, N6FA, LA, and PUFA with dementia risk and mortality between higher and lower AD-PRS groups. This suggests PUFAs' protective effects may overshadow genetic predisposition. However, a higher N6FA/N3FA ratio in the low AD-PRS group was linked to increased all-cause mortality, indicating that the balance of this ratio may drive interactions between PUFAs and genetic factors. Moreover, individuals with low-to-moderate *APOE* risk exhibited stronger protective associations of PUFA, N6FA, DHA, and LA intake against the onset of dementia, and from N3FA and DHA intake against dementia-related mortality, compared to those with higher *APOE* risk (*ε3/ε4* and *ε4/ε4*). This may be attributed to differences in the metabolic response and brain utilization of PUFAs among *APOE* genotypes [52]. Specifically, higher *APOE* risk levels may compromise blood-brain barrier function, thereby reducing the efficient transport and brain utilization of DHA and other PUFAs [53]. Moreover, individuals with higher dementia risk associated with *APOE* exhibit an elevated N6FA/N3FA ratio in the brain [54]. An increased N6FA/N3FA ratio has been associated with a higher likelihood of dementia and increased mortality. The neuroprotective effects of PUFAs may therefore be attenuated in individuals with higher *APOE* genetic risk due to altered lipid composition and impaired PUFA transport into the brain. Furthermore, higher *APOE* risk disrupts the metabolic coupling of fatty acids between neurons and astrocytes—reducing the efficiency of fatty acid transfer from neuronal lipid droplets and limiting astrocytic fatty acid oxidation [55]. This leads to lipid accumulation in the brain, contributing to dysregulated energy metabolism and increased oxidative stress. In addition, higher *APOE* risk is associated with impaired autophagy and mitochondrial dysfunction in astrocytes [56], which further exacerbate lipid and energy imbalance and promote tau and amyloid-β (Aβ) pathology.

Additionally, we observed that men with higher PUFA levels had lower dementia risk and higher survival rates compared to women. This gender difference may stem from variations in brain utilization of PUFAs and overall metabolic differences [57]. For instance, women have higher levels of PUFA metabolites (e.g. 13-HODE and 9-HODE) linked to neuroinflammation, potentially increasing dementia risk [58]. Additionally, hormonal changes, particularly the drop in estrogen levels after menopause, may also render older women more susceptible to dementia and less responsive to PUFA intake.

To the best of our knowledge, this is the first large prospective study examining the relationship between PUFA and dementia among hypertensive patients. Our findings highlight the potential health benefits of PUFA intake in reducing dementia incidence and mortality.

However, the present study has several limitations. First, the hypertensive participants in our study were predominantly White, which may limit the applicability of the results to more diverse populations. Second, plasma PUFA levels were measured only at baseline for a majority of individuals, preventing us from assessing longitudinal changes over time. Additionally, we aimed to prioritize robust, consistently measured, and widely available covariates to minimize misclassification and ensure model stability, however, residual confounding effects may still exist. For instance, possible confounders such as blood lipid profiles, inflammatory markers, baseline cognitive function, medication usage, or detailed dietary patterns were not included due to measurement limitations, potential mediator roles, or data unavailability in UK Biobank.

This study elucidates the interaction between PUFAs and genetic risk factors, particularly elevated *APOE* genetic risk, in the incidence and mortality of dementia, highlighting significant scientific and clinical implications. Scientifically, it enhances our understanding of the complex interplay between nutritional and genetic determinants in dementia pathogenesis, suggesting that increased *APOE* risk may diminish the neuroprotective effects of PUFAs and uncovering potential biological mechanisms underlying individual variability in response to PUFA-based interventions. From a clinical perspective, these findings offer valuable insights for identifying high-risk populations, such as individuals with higher *APOE* risk, and emphasize the importance of personalized PUFA supplementation strategies. By supporting the integration of genetic profiling into dementia prevention frameworks, this research facilitates the development of tailored nutritional recommendations and early intervention approaches in clinical practice. Future studies may investigate the effects of different forms of PUFAs—such as free DHA and phospholipid-bound DHA—in *APOE4* transgenic mice, uncovering specific mechanisms of the interaction between PUFAs and *APOE4*.

## 5 Conclusions

In this large prospective cohort study, we found that higher blood levels of DHA, N3FA, N6FA, LA, and total PUFA were linked to lower dementia incidence and mortality risk. Conversely, a higher N6FA/N3FA ratio was associated with increased risks. The protective associations of PUFAs were consistent across different PRS for AD and PUFAs. However, the protective associations of PUFA were more pronounced in those with low-to-moderate risk level associated with *APOE* status compared with those with higher *APOE* risk. These results highlight the importance of dietary fatty acid balance in reducing dementia risk, with potential for targeted nutritional strategies in hypertensive populations.

## Funding

This study was supported by National Nature Science Foundation of China (82,203,985).

## Data sharing

All data used in this study were obtained from the UK Biobank. Further information including the procedures to obtain and access data from the UK Biobank can be found at https://www.ukbiobank.ac.uk/. Code book, and analytic code used in this study can be made available upon reasonable request to the corresponding authors.

## CRediT authorship contribution statement

**Yubo Zhang:** Writing – review & editing, Writing – original draft. **Jindi Li:** Writing – review & editing, Visualization. **Shaohui Liu:** Validation, Formal analysis. **Quanhong Chen:** Investigation. **Xuexiu Wang:** Investigation. **Sisi He:** Investigation. **Yadong Wei:** Investigation. **Yunfeng Zou:** Supervision. **Yunan Xu:** Writing – review & editing, Methodology. **Lijun Wang:** Conceptualization. **Hao Chen:** Writing – review & editing, Supervision, Conceptualization.

## Declaration of competing interest

The authors declare that they have no known competing financial interests or personal relationships that could have appeared to influence the work reported in this paper.

## Appendix Supplementary materials

Image, application 1
