# Supplementary material for: Polyunsaturated fatty acids, APOE genotypes, and dementia incidence and mortality among hypertensive adults
Source: J Prev Alzheimers Dis. 2025 Jul 22;12(8):100297. doi: 10.1016/j.tjpad.2025.100297 (PMC12413715; doi:10.1016/j.tjpad.2025.100297)
Supplement: Supplementary file 1 [file mmc1.docx]

**Polyunsaturated fatty acids, *APOE* genotypes, and dementia incidence and mortality among hypertensive adults**

Yubo Zhang, BS^1,#^, Jindi Li, BS^2,#^, Shaohui Liu, MS^1^, Quanhong Chen, MS^2^, Xuexiu Wang, MS^1^, Sisi He, BS^1^, Yadong Wei, BS^1^, Yunfeng Zou, PhD^2,3,4^, Yunan Xu, PhD^5,*^, Lijun Wang, PhD^3,4,6*^, Hao Chen, PhD^1,3,4,*^

1. Department of Occupational and Environmental Health, School of Public Health,

Guangxi Medical University, Nanning, 530021, China

2. Department of Toxicology, School of Public Health, Guangxi Medical University,

Nanning, 530021, China

3. Department of Medical Research. The First Affiliated Hospital of Guangxi Medical University, Nanning, 530021, China

4. Department of Epidemiology and Biostatistics, School of Public Health, Guangxi Medical University, Nanning, Guangxi 530021, China

5. Guangxi Key Laboratory of Environment and Health Research, Guangxi Medical University, Nanning, Guangxi 530021, China

6. Guangxi Colleges and Universities Key Laboratory of Prevention and Control of Highly Prevalent Diseases, Guangxi Medical University, Nanning, 530021, China

^#^These authors contributed equally to this work.

^*^ Corresponding authors:

Dr. Hao Chen, Tel: +86 771 5358955, E-mail: [hchen255@gmail.com](mailto:hchen255@gmail.com).

Dr. Lijun Wang, Tel: +86 771 5358955, E-mail: [wanglj1003@163.com](mailto:wanglj1003@163.com).

Dr. Yunan Xu, Tel: +86 771 5356557, E-mail: [xuyunan3175@163.com](mailto:xuyunan3175@163.com).

Table S1. Associations between the blood levels of PUFA and the incidence and mortality of dementia among hypertensive adults in the UK Biobank.

|  | Quartiles of the blood levels of polyunsaturated fatty acids | | | |  |
| --- | --- | --- | --- | --- | --- |
| Variables | Quartile 1 | Quartile 2 | Quartile 3 | Quartile 4 | *P*-trend* |
| Incidence of dementia |  |  |  |  |  |
| PUFA | 1.00 | 0.83(0.76,0.91) *** | 0.79(0.72,0.87) *** | 0.71(0.64,0.78) *** | <0.001 |
| N3FA | 1.00 | 0.92(0.83,1.01) | 0.83(0.75,0.92) *** | 0.77(0.70,0.85) *** | <0.001 |
| N6FA | 1.00 | 0.86(0.78,0.94) ** | 0.79(0.72,0.87) *** | 0.72(0.65,0.80) *** | <0.001 |
| N6FA/N3FA | 1.00 | 1.15(1.05,1.26) ** | 1.14(1.03,1.26) ** | 1.22(1.10,1.35) *** | <0.001 |
| DHA | 1.00 | 0.93(0.85,1.03) | 0.80(0.72,0.88) *** | 0.78(0.71,0.86) *** | <0.001 |
| LA | 1.00 | 0.89(0.81,0.97) * | 0.78(0.71,0.86) *** | 0.74(0.67,0.82) *** | <0.001 |
| Dementia mortality |  |  |  |  |  |
| PUFA | 1.00 | 0.74(0.60,0.92) ** | 0.78(0.63,0.97) * | 0.58(0.45,0.73) *** | <0.001 |
| N3FA | 1.00 | 0.86(0.69,1.06) | 0.62(0.50,0.78) *** | 0.65(0.52,0.82) *** | <0.001 |
| N6FA | 1.00 | 0.74(0.60,0.92) ** | 0.75(0.60,0.94) * | 0.61(0.48,0.77) *** | <0.001 |
| N6FA/N3FA | 1.00 | 1.00(0.81,1.25) | 1.25(1.01,1.55) * | 1.34(1.07,1.67) * | 0.003 |
| DHA | 1.00 | 0.93(0.75,1.15) | 0.69(0.55,0.87) ** | 0.71(0.57,0.89) ** | <0.001 |
| LA | 1.00 | 0.82(0.66,1.01) | 0.80(0.64,1.00) * | 0.70(0.56,0.89) ** | 0.004 |
| All-cause mortality |  |  |  |  |  |
| PUFA | 1.00 | 0.80(0.76,0.84) *** | 0.71(0.68,0.75) *** | 0.66(0.62,0.69) *** | <0.001 |
| N3FA | 1.00 | 0.79(0.76,0.83) *** | 0.71(0.68,0.74) *** | 0.64(0.61,0.67) *** | <0.001 |
| N6FA | 1.00 | 0.82(0.78,0.86) *** | 0.73(0.70,0.77) *** | 0.70(0.66,0.73) *** | <0.001 |
| N6FA/N3FA | 1.00 | 1.09(1.04,1.15) *** | 1.18(1.13,1.24) *** | 1.43(1.36,1.50) *** | <0.001 |
| DHA | 1.00 | 0.80(0.76,0.83) *** | 0.71(0.68,0.74) *** | 0.62(0.59,0.66) *** | <0.001 |
| LA | 1.00 | 0.84(0.80,0.88) *** | 0.75(0.72,0.79) *** | 0.70(0.66,0.73) *** | <0.001 |

Note: This model utilized the cox proportional hazards model. The model was adjusted for covariates including age, sex/gender, race/ethnicity, educational level, annual household income, body mass index, smoking status, alcohol use in the past year, physical activity level, diabetes, cardiovascular disease, sleep duration, Townsend deprivation index, taking antihypertensive medicine, and *APOE* genotypes. *APOE*: apolipoprotein E, DHA: docosahexaenoic acid, LA: linoleic acid, N3FA: omega-3 polyunsaturated fatty acids, N6FA: omega-6 polyunsaturated fatty acids, PUFA: polyunsaturated fatty acids. * *p* < 0.05; ** *p* < 0.01; *** *p* < 0.001.

Table S2. Gender-stratified analysis of the association between the blood levels of PUFAs and the incidence and mortality of dementia among hypertensive adults in the UK Biobank.

|  | Hazards ratio [95%CI] | |  |
| --- | --- | --- | --- |
|  | Men | Women | *P*-value |
| Dementia incidence |  |  |  |
| PUFA | 0.82(0.77,0.87) *** | 0.92(0.86,0.98) * | 0.03 |
| N3FA | 0.59(0.46,0.74) *** | 0.86(0.68,1.08) | 0.02 |
| N6FA | 0.80(0.74,0.86) *** | 0.91(0.84,0.98) * | 0.03 |
| N6FA/N3FA | 1.02(1.00,1.02) *** | 1.01(1.00,1.02) | 0.03 |
| DHA | 0.33(0.18,0.60) *** | 0.77(0.42,1.41) | 0.04 |
| LA | 0.80(0.74,0.86) *** | 0.91(0.84,0.98) * | 0.03 |
| Dementia mortality |  |  |  |
| PUFA | 0.81(0.70,0.94) ** | 0.85(0.73,0.99) * | 0.85 |
| N3FA | 0.51(0.29,0.87) * | 0.71(0.42,1.19) | 0.43 |
| N6FA | 0.81(0.68,0.96) * | 0.83(0.70,1.00) * | 0.99 |
| N6FA/N3FA | 1.02(1.00,1.04) * | 1.02(1.00,1.04) | 0.77 |
| DHA | 0.28(0.07,1.15) | 0.50(0.12,2.04) | 0.62 |
| LA | 0.83(0.70,0.99) * | 0.83(0.70,0.99) * | 0.86 |
| All-cause mortality |  |  |  |
| PUFA | 0.86(0.84,0.88) *** | 0.88(0.84,0.91) *** | 0.32 |
| N3FA | 0.49(0.44,0.55) *** | 0.63(0.55,0.72) *** | <0.01 |
| N6FA | 0.87(0.84,0.90) *** | 0.88(0.84,0.92) *** | 0.71 |
| N6FA/N3FA | 1.02(1.02,1.03) *** | 1.01(1.01,1.02) *** | <0.01 |
| DHA | 0.17(0.13,0.23) *** | 0.25(0.17,0.35) *** | 0.08 |
| LA | 0.88(0.85,0.91) *** | 0.88(0.85,0.92) *** | 0.73 |

Note: This model utilized the cox proportional hazards model. The model was adjusted for covariates including age, sex/gender, race/ethnicity, educational level, annual household income, body mass index, smoking status, alcohol use in the past year, physical activity level, diabetes, cardiovascular disease, sleep duration, Townsend deprivation index, taking antihypertensive medicine, and *APOE* genotypes. *APOE*: apolipoprotein E, DHA: docosahexaenoic acid, LA: linoleic acid, N3FA: omega-3 polyunsaturated fatty acids, N6FA: omega-6 polyunsaturated fatty acids, PUFA: polyunsaturated fatty acids, PRS: polygenic risk scores. * *p* < 0.05; ** *p* < 0.01; *** *p* < 0.001.

Table S3. Sensitivity analysis of the association between the blood levels of PUFAs and dementia incidence and mortality among hypertensive adults in the UK Biobank.

|  | Hazards ratio [95%CI] |
| --- | --- |
| Dementia incidence |  |
| PUFA | 0.85(0.82,0.89) *** |
| N3FA | 0.66(0.57,0.77) *** |
| N6FA | 0.84(0.80,0.88) *** |
| N6FA/N3FA | 1.01(1.01,1.02) *** |
| DHA | 0.40(0.27,0.60) *** |
| LA | 0.84(0.80,0.88) *** |
| Dementia mortality |  |
| PUFA | 0.80(0.72,0.88) *** |
| N3FA | 0.53(0.37,0.76) *** |
| N6FA | 0.78(0.69,0.88) *** |
| N6FA/N3FA | 1.02(1.01,1.03) *** |
| DHA | 0.26(0.10,0.67) ** |
| LA | 0.80(0.71,0.90) *** |
| All-cause mortality |  |
| PUFA | 0.81(0.79,0.83) *** |
| N3FA | 0.46(0.42,0.49) *** |
| N6FA | 0.81(0.79,0.83) *** |
| N6FA/N3FA | 1.02(1.02,1.02) *** |
| DHA | 0.11(0.09,0.14) *** |
| LA | 0.82(0.80,0.85) *** |

Note: This model utilized the cox proportional hazards model. The model was adjusted for covariates including age, sex/gender, race/ethnicity, educational level, annual household income, body mass index, smoking status, alcohol use in the past year, physical activity level, diabetes, cardiovascular disease, sleep duration, Townsend deprivation index, taking antihypertensive medicine, and *APOE* genotypes. *APOE*: apolipoprotein E, DHA: docosahexaenoic acid, LA: linoleic acid, N3FA: omega-3 polyunsaturated fatty acids, N6FA: omega-6 polyunsaturated fatty acids, PUFA: polyunsaturated fatty acids. * *p* < 0.05; ** *p* < 0.01; *** *p* < 0.001.


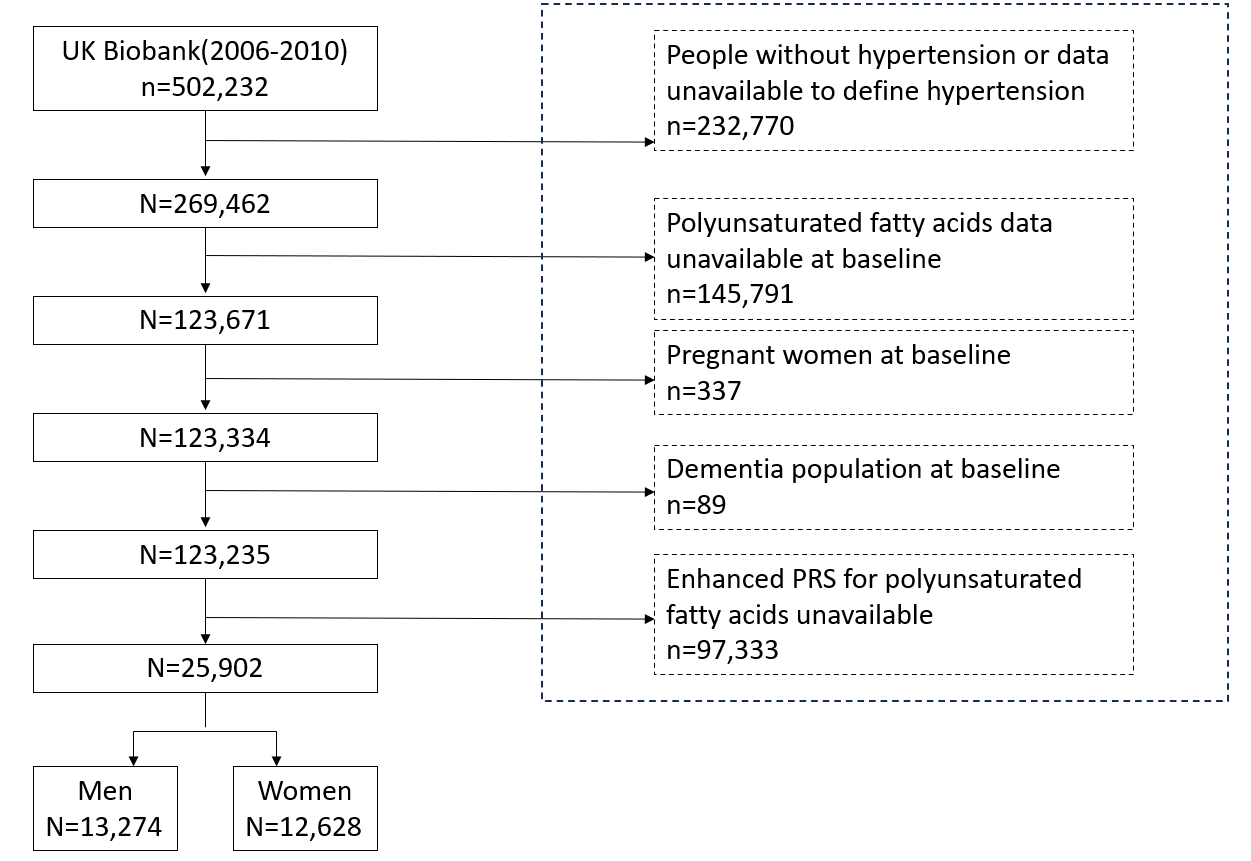


Figure S1. Flow chart of sample selection


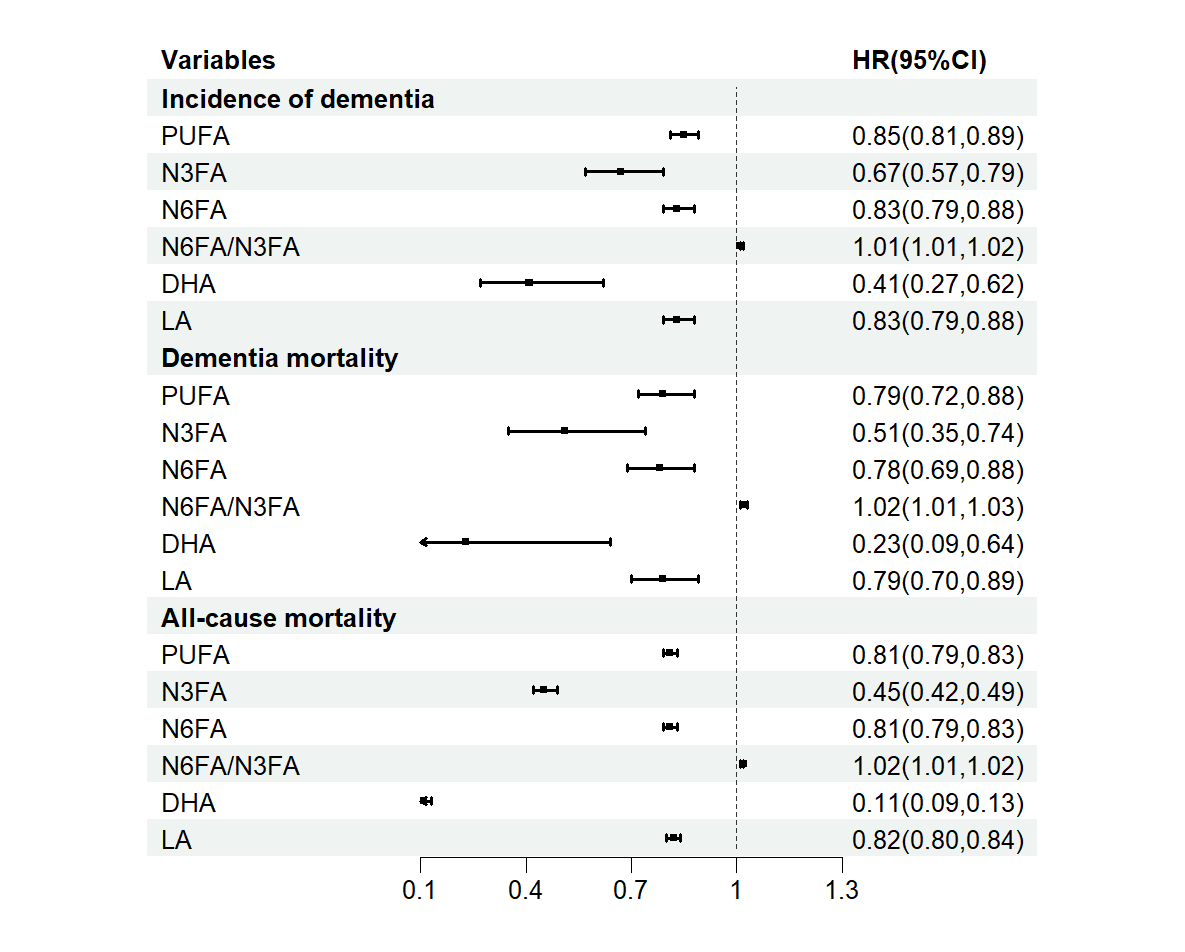


Figure S2. Sensitivity analysis of the associations between PUFAs and dementia and mortality risk among hypertensive populations in the UK Biobank additionally controlling for depression status.

Note: A cox proportional hazards model was utilized for the analysis. The model was adjusted for covariates including age, sex/gender, race/ethnicity, educational level, annual household income, body mass index, smoking status, alcohol use in the past year, physical activity level, diabetes, cardiovascular disease, sleep duration, Townsend deprivation index, taking antihypertensive medicine, *APOE* genotypes, and depression status. *APOE*: apolipoprotein E, DHA: docosahexaenoic acid, LA: linoleic acid, N3FA: omega-3 polyunsaturated fatty acids, N6FA: omega-6 polyunsaturated fatty acids, PUFA: polyunsaturated fatty acids.


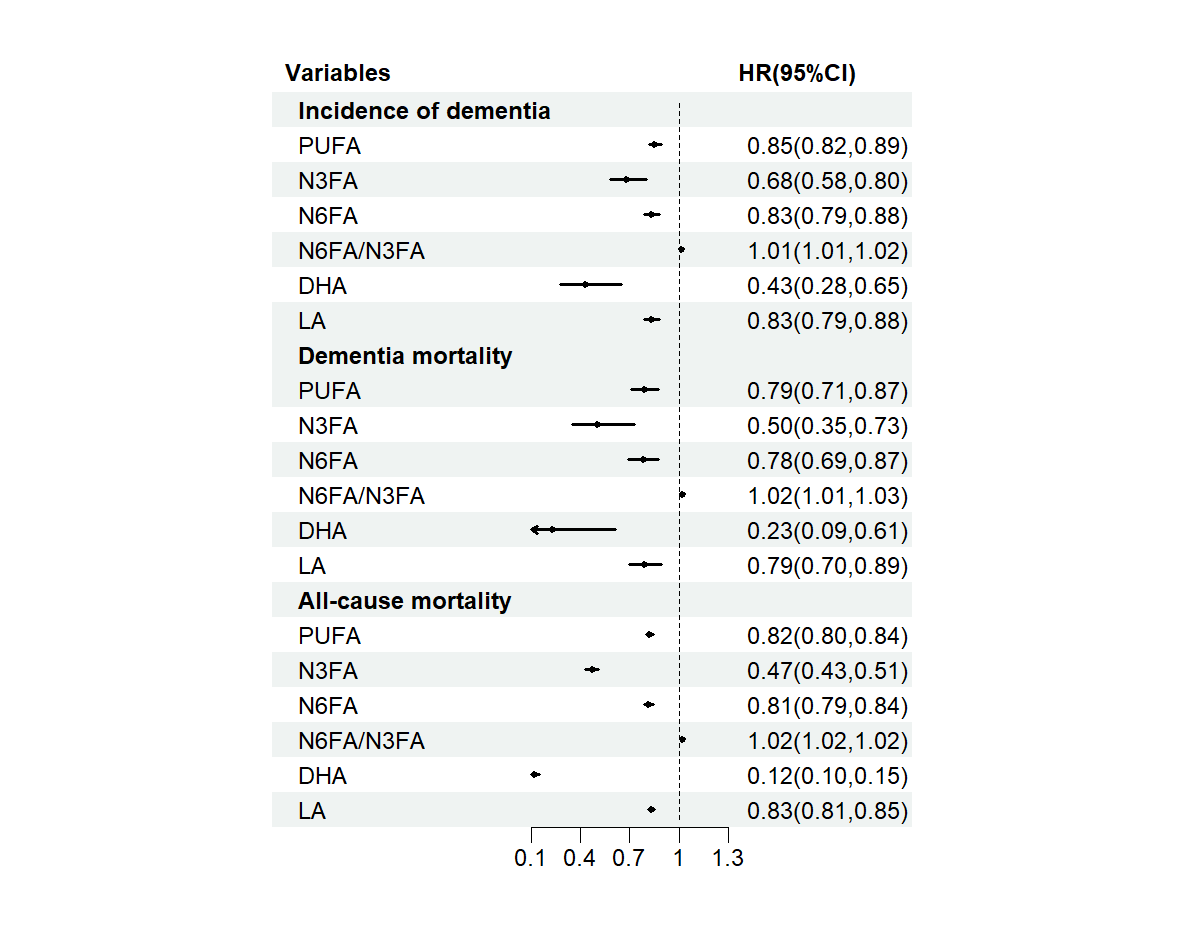


Figure S3. Sensitivity analysis of the associations between PUFAs and dementia and mortality risk among hypertensive populations in the UK Biobank with dementia incidence and death cases occurred two years post baseline.

Note: This model utilized the cox proportional hazards model. The model was adjusted for covariates including age, sex/gender, race/ethnicity, educational level, annual household income, body mass index, smoking status, alcohol use in the past year, physical activity level, diabetes, cardiovascular disease, sleep duration, Townsend deprivation index, taking antihypertensive medicine and *APOE* genotypes. Only cases who developed disease or died two years after baseline enrollment were included for the analysis. *APOE*: apolipoprotein E, DHA: docosahexaenoic acid, LA: linoleic acid, N3FA: omega-3 polyunsaturated fatty acids, N6FA: omega-6 polyunsaturated fatty acids, PUFA: polyunsaturated fatty acids.
